# The Nature of Preparations Used in the Treatment of Drug Addictions

**Published:** 1909-08

**Authors:** 


The Nature of Preparations Used in the Treatment of Drug Addictions.—Dr. L. F. Kebler, of Washington, said that examinations by the Department of Agriculture during the past few years had shown that preparations used for the treatment of drug addictions usually contained large quantities of the drug for which treatment was instituted. He cited examples in which he had found preparations containing as much as thirty-two grains of morphine to the fluid ounce. Sometimes they contained also atropine, strychnine, quinine, or other substances. In some cases there was a gradual reduction in successive supplies of the preparation, but the reduction was so small as virtually to extend the treatment over years. In other cases, the treatment was with packages serially numbered from one to ten and each package was found to contain the same amount of the habit forming drug. The books of some institutions showed that the treatment was intended to be continued indefinitely. These practices were exceedingly difficult to control, but the Postoffice Department had been of the greatest assistance, and it was hoped that it would be successful in eliminating them entirely.—*New York Medical Journal.*

